# lncRNA HOXC-AS2 promotes the progression of hypopharyngeal cancer by binding to the P62 protein mediating the autophagy process

**DOI:** 10.18632/aging.205192

**Published:** 2023-11-08

**Authors:** Yuandi Xiang, Guoquan Huang, Jie Wang, Qingquan Hua

**Affiliations:** 1 Department of Otolaryngology-Head and Neck Surgery, Renmin Hospital of Wuhan University, Wuhan, Hubei 430060, China; 2 Hubei Selenium and Human Health Institute, The Central Hospital of Enshi Tujia and Miao Autonomous Prefecture, Enshi, Hubei 445000, China; 3 Hubei Provincial Key Lab of Selenium Resources and Bioapplications, Enshi, Hubei 445000, China; 4 Department of Gastrointestinal Surgery, The Central Hospital of Enshi Tujia and Miao Autonomous Prefecture, Enshi, Hubei 445000, China

**Keywords:** HOXC-AS2, hypopharyngeal cancer, P62, autophagy

## Abstract

Hypopharyngeal carcinoma is the most malignant type of head and neck squamous cell carcinoma, and lncRNAs play an important role in its formation and progression. However, the related specific mechanisms are rarely studied. lncRNAs closely associated with hypopharyngeal cancer were examined by lncRNA sequencing for in-depth exploration of the relationship between HOXC-AS2 and hypopharyngeal cancer pathogenesis. The mRNA expression of HOXC-AS2 and related genes was measured by qRT-PCR, and the biological function of HOXC-AS2 in hypopharyngeal carcinoma was demonstrated by gain- and loss-of-function experiments. RNA pulldown, RNA immunoprecipitation (RIP) and gene body truncation experiments and transcriptome sequencing were used to investigate the potential mechanism of HOXC-AS2 and its downstream genes, including P62, NF-KB and HMOX1. Finally, the biological function of HOXC-AS2 was confirmed in animal experiments. HOXC-AS2 and P62 expression was significantly upregulated in hypopharyngeal cancer tissues compared with normal hypopharyngeal tissues, while HMOX1 expression was decreased. Functionally, HOXC-AS2 overexpression can promote the viability, proliferation, migration and invasion of hypopharyngeal cancer cells and facilitate hypopharyngeal cancer progression. It was confirmed that HOXC-AS2 can bind to the P62 protein and activate the NF-KB signaling pathway, thereby affecting HMOX1 expression and regulating autophagy in hypopharyngeal cancer cells, ultimately regulating the formation and progression of hypopharyngeal cancer. In conclusion, our findings suggest that HOXC-AS2 regulates the progression of hypopharyngeal cancer by regulating autophagy and is abnormally highly expressed in hypopharyngeal cancer tissues. HOXC-AS2 may become a new target for the diagnosis and treatment of hypopharyngeal cancer.

## INTRODUCTION

Hypopharyngeal squamous cell carcinoma (HSCC) is a malignant tumor with the worst prognosis among head and neck squamous cell carcinomas (HNSCCs). In recent years, its incidence rate has risen rapidly [1]. At the time of diagnosis, most HSCCs are in stage III or IV, and the 5-year overall survival rate is less than 45% [2]. Over the past decades, researchers worldwide have made many efforts to increase the survival time and improve the quality of life of hypopharyngeal cancer patients. Although surgery, radiotherapy, chemotherapy and molecular targeted therapy have been applied in the clinical treatment of hypopharyngeal cancer, the 5-year overall survival rate of patients with hypopharyngeal cancer has not been significantly increased, and half of the patients still do not achieve the ideal effect [3]. Therefore, the in-depth study of the mechanism of hypopharyngeal cancer occurrence and development, identification of markers that can predict the progress and prognosis of the disease, and exploration of new therapeutic targets are key to increasing the survival time and improving the quality of life of patients with hypopharyngeal cancer.

Long noncoding RNAs (lncRNAs) are noncoding transcripts produced during transcription that regulate gene expression and protein function [4, 5]. They do not have intrinsic protein-coding ability, but they can regulate the expression of oncogenes or tumor suppressor genes by binding to RNA-binding proteins, functioning as miRNA decoys, participating in selective splicing of mRNAs, and mediating epigenetic and posttranslational regulation [6–9], and they play important roles in the occurrence and development of tumors. Recent studies have shown that lncRNAs are abnormally expressed in various types of cancer, including hypopharyngeal cancer [10–12]. Our research group collected 3 matched early-stage HSCC primary foci and normal control tissues. Through lncRNA sequencing and bioinformatics analysis, we found that 140 lncRNAs were significantly upregulated and 138 lncRNAs were significantly downregulated. Among the differentially expressed lncRNAs, HOXC-AS2 exhibited the highest expression. Recent studies have shown that HOXC-AS2 is closely related to the progression and metastasis of endometrial cancer [13] and non-small cell lung cancer (NSCLC) [14] through a competing endogenous RNA (ceRNA) mechanism. To date, the mechanism of action of HOXC-AS2 in hypopharyngeal carcinoma has not been studied.

lncRNAs can bind to proteins and play influential roles. Through an RNA pulldown experiment, we found that HOXC-AS2 can bind to the Sequestosome-1 (SQSTM2/P 62) protein. SQSTM1/p62 is an important selective autophagy adaptor protein. It contains six functional domains, including a ubiquitin-related domain; a Kelch-like -ECH-associated protein domain; a microtubule-related protein 1A/1B light chain 3 domain; a tumor necrosis factor receptor-related factor 6, Phox and Bem1p domain; and a zinc finger domain [15]. SQSTM1/p62 plays an important role in the clearance of ubiquitinated proteins, and its abnormal expression is closely related to the occurrence and development of many tumors and chronic diseases. Current studies have shown that P62 can regulate the formation, development and metastasis of hypopharyngeal cancer and lung cancer [15, 16]. The multifunctional structure of p62 makes it the hub of multiple signaling pathways involved in the maintenance and regulation of basic cell functions. Therefore, abnormal function of p62/SQSTM1 can lead to blockade of various signaling pathways and abnormal protein aggregation, thus leading to the development of various diseases. Current studies have confirmed that autophagy defects in a variety of tumor cells, accompanied by robust p62 accumulation, are related to the regulation of NF-κB [17, 18]. However, NF-KB is regulated by related lncRNAs in hypopharyngeal cancer, and this regulation has not yet been studied and needs further exploration.

Therefore, this study aims to study the expression and clinical significance of HOXC-AS2 in HSCC, explore its correlation with the clinical characteristics of patients with HSCC, and investigate the potential mechanism of HOXC-AS2 in HSCC through *in vivo* and *in vitro* experiments. Our results show that HOXC-AS2 can be used as a diagnostic marker for and a potential therapeutic target in hypopharyngeal cancer.

## MATERIALS AND METHODS

### Clinical tissue samples and patients

The clinical samples in this study included 3 fresh sequencing samples and 16 paraffin samples. The three fresh sequencing samples, which included cancerous tissue and adjacent normal tissue (obtained from a region at least approximately 1.0 cm from the edge of the cancerous tissue and demonstrating a negative result on the intraoperative rapid frozen section test), were obtained from the Provincial People’s Hospital of Wuhan University (Supplementary Table 1). The 16 paraffin samples were obtained from the Central Hospital of Enshi Tujia and Miao Autonomous Prefecture of Hubei Province. All patients were diagnosed with HSCC according to the American Joint Committee on Cancer (AJCC) tumor staging manual and histopathological evaluation [19]. None of the patients received preoperative radiotherapy or chemotherapy. Informed consent was obtained from each patient, and the study was approved by the ethics committees of Renmin Hospital of Wuhan University and Enshi Tujia and Miao Autonomous Prefecture of Hubei Province (Wuhan, China; Ethics. Enshi, China: Ethics Approval Number: 2023-062-001).

### lncRNA sequencing of hypopharyngeal carcinoma primary lesions and adjacent normal tissues

The differentially expressed lncRNAs between hypopharyngeal carcinoma and adjacent normal tissues were identified through lncRNA sequencing (Singleron Bio Com, Nanjing, China). The main steps were as follows: Total RNA was extracted from the samples with TRIzol. After total RNA was obtained, its concentration and purity were determined, and its integrity was confirmed by RNA-specific agarose electrophoresis. rRNA was removed with an rRNA removal kit. The RNA was then fragmented into segments of approximately 200-300 bp by ionic disruption, and then the segments with a length of 300 bp were selected. cDNA was synthesized by reverse transcription with random primers. First-strand cDNA was synthesized with 6-base random primers and reverse transcriptase using RNA as the template, and second-strand cDNA was synthesized with the first-strand cDNA as the template. After construction of the library, PCR amplification was used to enrich the library fragments, and the library was then screened according to the fragment size. The library size was 450 bp. Then, the library was assessed by an Agilent 2100 Bioanalyzer, and the total concentration and effective concentration of the library were determined. Next, based on the effective concentration of the library and the amount of data required, the library containing different index sequences was proportionally mixed. The mixed library was uniformly diluted to 2 nM, and the single-stranded library was generated by alkaline denaturation. Finally, these libraries were subjected to next-generation sequencing (NGS) on the Illumina sequencing platform.

### Cell lines and culture

The hypopharyngeal cancer cell lines used in this study were FADU and Detroit-562, which were purchased from Jinyuan Biotechnology Co., Ltd., (Shanghai, China) and Procell (Wuhan, China), respectively. The FADU cell line was cultured using DMEM (Gibco, USA) containing 10% fetal bovine serum (FBS) (Gibco, USA), and the Detroit-562 cell line was cultured using a dedicated medium (Procell, Wuhan, China). All cell lines were incubated in an incubator with 5% CO_2_ at 37°C. When the cultured cells were 90–95% confluent, they were detached from the culture flask or culture dish with trypsin (0.25%) (Gibco, USA). The cells were then subcultured and frozen, and subsequent experiments were performed.

### RNA extraction and quantitative reverse transcription-polymerase chain reaction (qRT-PCR)

For cell or tissue samples, total RNA was extracted by using the EASYspin Tissue and Cellular RNA Rapid Extraction Kit (Aidlab, Beijing, China) according to the manufacturer’s protocol and RNA extraction for the immunoprecipitation experiments was performed with TRIzol Reagent (Invitrogen, USA). The specific steps were performed strictly according to the instructions provided by the reagent manufacturers. Then, the total RNA concentration was measured using a full-spectrum spectrophotometer (NanoDrop 2000, Thermo Fisher Scientific, USA), and total RNA was reverse transcribed into cDNA according to the manufacturer’s protocol (Vazyme, Nanjing, China). Next, we carried out qRT-PCR on a PCR system (IQ5, Bio-Rad, USA) utilizing SYBR-Green PCR Master Mix (Vazyme, Nanjing, China) in a 20 μl reaction system. The relative expression of the target genes was calculated using the 2−ΔΔCt method, and all primers and their sequences used in this study are listed in Supplementary Table 2. Finally, a histogram was constructed by utilizing special software (GraphPad Prism 8, Graphpad Software Inc., USA) after the corresponding expression values were obtained.

### Subcellular fractionation and RNA fluorescence in situ hybridization (FISH)

#### Subcellular fractionation

FADU and Detroit-562 cells were cultured, collected, and washed three times with ice-cold PBS. Next, we performed nuclear and cytosolic fractionation by utilizing the Nuclear and Cytoplasmic Extraction Kit (PARISTM-AM1921, Thermo Fisher Scientific, USA) according to the manufacturer’s instructions. Then, total RNA was extracted from cytoplasmic and nuclear samples using a rapid RNA extraction kit (Aidlab, Beijing, China). After the concentration of total RNA was measured, total RNA was reverse transcribed into cDNA. Finally, the relative expression of HOXC-AS2 in the cytoplasm and nucleus was determined through qRT-PCR analysis, and β-actin and U6 were used as reference genes for the cytoplasm and nucleus, respectively. Histograms of relative expression were generated using GraphPad Prism software (GraphPad Prism 8, Graphpad Software Inc., USA).

#### FISH

The HOXC-AS2 FISH probe used in this study was designed and synthesized by GenePharma (Suzhou, China), and the probe sequence is detailed in the Supplementary Table 2. Based on the instructions for the FISH experiment, first, slides containing FADU and Detroit-562 cells were prepared. After qualification by microscopy, the cells were fixed with 4% paraformaldehyde. Next, FISH was performed; in brief, the cells were first treated with proteinase K (20 μg/mL) for 30 min and then washed three times with PBS prior to prehybridization, hybridization (probe hybridization solution concentration: 5–8 μM), and DAPI counterstaining of nuclei. Finally, the FISH results were obtained, and the slides were analyzed for positive staining under a fluorescence microscope (Nikon, Japan).

### Lentivirus, plasmids, siRNA and cell transfection

The overexpression and knockdown lentiviruses or plasmids used in this study, including those for HOXC-AS2 and SQSTM1, were designed, constructed, synthesized, sequenced and verified by Shanghai GeneChem Co., Ltd., (Shanghai, China). The detailed sequences are shown in Supplementary Table 2. The HOXC-AS2 overexpression lentivirus was named LV-Oe-HOXC-AS2, and the HOXC-AS2 knockdown lentivirus was named LV-SH-HOXC-AS2. FADU and Detroit-562 cells were transfected utilizing Lipofectamine 3000 (Invitrogen, USA) according to the manufacturer’s protocol. After 24 or 48 h of transfection, the cells were subjected to subsequent functional experiments or harvested for RNA or protein extraction and analysis. To construct stably transfected cell lines, lentiviruses, viral infection enhancer, and polybrene (final concentration 7 μg/mL, Sigma-Aldrich, USA) were first added to 24-well plates when the cells were approximately 40% confluent. Fresh DMEM containing 10% FBS was added 16 h after lentiviral infection and was replaced with medium containing the corresponding antibiotics 48 h later. Finally, the stably transduced cell lines were further selected with puromycin (Sigma-Aldrich, USA) and progressively transferred to 12-well plates, 6-well plates and culture flasks for culture. The fluorescence intensity of the infected cells was evaluated, and qRT-PCR or western blot (WB) analysis was subsequently performed.

### Western blot analysis and reagents

Hypopharyngeal cancer cells that met the experimental requirements were cultured and collected. Then, the cells were washed three times with PBS before the addition of RIPA buffer containing protease inhibitors (Thermo Fisher Scientific, USA). The assay concentration was determined according to the number of cells. Cells were lysed on ice for 30 min and were then subjected to sonication (AMP: 40%, 2 s, 3 s, 5 min), and the extracted proteins were quantified using the BCA method. In this study, proteins were separated through sodium dodecyl sulfate-polyacrylamide gel electrophoresis (SDS-PAGE) and then electrophoretically transferred to polyvinylidene difluoride (PVDF) membranes (Millipore, USA). Next, the membranes were blocked with nonfat milk (5%) for 1.5–2.0 h and incubated with primary antibodies overnight on a shaker in a refrigerator at 4°C. The concentration of the primary antibody was determined according to the generic antibody protocol. The primary antibody was removed by washing with TBST solution (5 min each, 5 times), and then a horseradish peroxidase (HRP)-conjugated secondary antibody was added for a 1 h incubation at room temperature. Finally, the target protein was visualized by chemiluminescence (Bio-Rad ChemiDoc XRS System, USA), and the expression of the target protein was analyzed through software (Bio-Rad Image Lab, USA). In our study, the primary antibodies and dilution ratios were as follows: anti-SQSTM1 (1:1000, Abcam, USA), anti-NF-kB (1:1000, CST, USA), anti-HMOX1 (1:1000, Santa Cruz, USA), and anti-GAPDH (1:5000, Santa Cruz, USA). The secondary antibodies were diluted 1:5000.

### Cell counting Kit-8 (CCK-8) assay

In this study, to detect the changes in the viability of hypopharyngeal cancer cells after overexpression or knockdown of HOXC-AS2, a CCK-8 assay was performed. According to the instructions of the CCK-8 kit, stable hypopharyngeal cancer cell lines with HOXC-AS2 overexpression or knockdown were first cultured, and the corresponding plasmids were transfected according to the experimental requirements for the rescue experiments. Then, the absorbance of the wells of hypopharyngeal cancer cells was measured at 0 h, 24 h, 48 h, 72 h, and 96 h, the difference between the groups was analyzed, and scatter plots were generated by GraphPad Prism software (GraphPad Prism 8, Graphpad Software Inc., USA).

### Colony formation assay

The proliferation ability of hypopharyngeal cancer cells after overexpression or knockdown of HOXC-AS2 was evaluated by a colony formation assay. In brief, stable FADU and Detroit-652 cell lines were seeded into 6-well plates (1000–1500 cells per well). The cells were cultured in the incubator for 14 days. Culture was terminated when the cell mass was macroscopically visible. After washing the cells with PBS three times, they were fixed with 4% paraformaldehyde, stained with 0.5% crystal violet, and then photographed under a microscope (Olympus-IX73, Olympus, Tokyo, Japan).

### Wound healing assay

The migration ability of HOXC-AS2-overexpressing and HOXC-AS2-knockdown hypopharyngeal cancer cells was evaluated by a wound healing assay. Briefly, stable hypopharyngeal cancer cell lines (FADU and Detroit-562) were seeded into 6-well plates. For rescue experiments, the corresponding plasmids were transfected into the stable cell lines. When the cells were 85–90% confluent, three wounds were created on the cell surface with a yellow pipette tip, and the cell debris was removed by washing with PBS. Next, we added serum-free medium and incubated the cells in an incubator at 37°C, photographing the same areas every 24 h to monitor wound healing. Finally, photographs were taken with a microscope (Olympus-IX73, Olympus, Tokyo, Japan), and the areas of the artificially created wounds were measured using ImageJ software (NIH, USA).

### Transwell migration and invasion assays

Transwell migration and invasion assays were used to evaluate the migration and invasion abilities of hypopharyngeal cancer cells after HOXC-AS2 overexpression or knockdown. Briefly, 24-well plates (8 μm pore size) were used; 1.50 × 105 cells were resuspended in 500 μL of serum-free DMEM and added to the upper chambers, and 750 μL of DMEM containing 10% FBS was added to the lower chambers. After 48 h of incubation, the cells were washed three times with PBS. Cells on the upper surface of the membrane in the upper chamber with a cotton swab, and cells on the lower surface of the membrane were fixed with 4% paraformaldehyde and stained with crystal violet (0.5%). Finally, five fields of view were selected under a microscope (Olympus-IX73, Olympus, Tokyo, Japan) for photography and cell counting. All experiments were repeated three times.

### RNA pulldown assay

Based on the RNA Protein Pull-Down Kit (20164, Thermo Fisher Scientific, USA) protocol and the gene sequence of HOXC-AS2, a biotin-labeled full-length RNA probe was designed and synthesized. Detailed sequences are provided in Supplementary Table 2. RNA pulldown assays were carried out with the FADU cell line. In brief, throughout the experiment, it was necessary to keep the work area nuclease-free, strictly clean and sterilized; wear gloves; and use only reagents and plastics compatible with nucleic acids. In brief, the steps were as follows: wash the streptavidin magnetic beads; prepare the experimental cell lysates; label the target RNA by utilizing the RNA 3′ Desthiobiotinylation Kit; bind HOXC-AS2 to streptavidin magnetic beads with a biotin-labeled RNA probe; and bind RNA-binding proteins (RBPs) to RNA. In this step, after the addition of RNA-bound beads, the mixture was incubated for 1 h in a 4°C refrigerator with rotation; then, the RNA-binding protein complexes were washed using 1× wash buffer (100 μL), and elution buffer was added to the protein products and incubated for 30 min at 37°C with rotation. Finally, the products were heated for 10 min at 100°C, and western blot analysis and mass spectrometry (MS) were performed. After the MS results were obtained, we performed bioinformatics analysis and then identified the proteins associated with HOXC-AS2. After verification, we carried out subsequent experiments.

### Coimmunoprecipitation (co-IP) assay

According to the co-IP Kit protocol (Abison, Guangzhou, China), hypopharyngeal cancer cells with overexpression or knockdown were cultured. After removing the medium, the cells were washed 3 times with PBS, and 0.5 ml ice-cold lysis buffer was then added to the cell culture dish for incubation on ice for 5 min. Next, we collected and transferred the samples to new Eppendorf (EP) tubes. Then, the co-IP assay was performed strictly according to the instructions. The obtained products were subjected to western blot analysis to confirm protein interactions or subjected to MS, after which the interacting proteins were identified by bioinformatics analysis.

### Transmission electron microscopy

D-562 cells with stable HOXC-AS2 knockdown were cultured, and the negative control (NC)-expressing group was used as the control group. Then, cells were collected and prepared as required for transmission electron microscopy to evaluate the changes in autophagosomes in D-562 cells after HOXC-AS2 knockdown.

### Animal experiments

The animal experiments were approved by the Ethics Committee of Hubei Provincial People’s Hospital of Wuhan University. Four- to six-week-old mice were purchased from Beijing Vital River Laboratory Animal Technology Co., Ltd. The mice were divided into four groups: the lentivirus-mediated HOXC-AS2 knockdown negative control (LV-KDNC-HOXC-AS2) and lentivirus-mediated knockdown of HOXC-AS2 (KD-HOXC-AS2) groups and the LV-KD-HOXC-AS2+OE-SQSTM1 and LV-KD-HOXC-AS2 groups (*n* = 5 mice per group). Then, Detroit-562 cells were cultured according to the experimental group design, and the cells (6 × 10^5^) were resuspended in 100 μl of DMEM and injected into the flanks of mice. Two weeks later, tumor formation was observed and recorded, and the tumor volume was calculated using the following equation: volume = 1/2 × (width2 × length). Five weeks later, the mice were euthanized, and we collected subcutaneous tumors for further experiments.

### Statistical analysis

In this study, we used GraphPad Prism (version 8.0, GraphPad Software Inc., USA) and SPSS software (version 23.0, IBM SPSS, USA) to analyze our data. The relationship between HOXC-AS2 and SQSTM1 protein expression was evaluated through Pearson correlation analysis. The chi-square test was used to analyze the relationships between the expression levels of HOXC-AS2 and SQSTM1 and the clinicopathological data of patients with hypopharyngeal carcinoma. A *t*-test, the Wilcoxon test or Fisher’s exact test was used for comparisons between the two groups. Analysis of variance or the chi-square test was used for comparisons of normally distributed data between groups. All cell experiments were performed independently at least three times. In all cases, differences were considered statistically significant when *P* < 0.05.

### Data availability

The data used to support the findings of this study are available from the corresponding author upon request.

## RESULTS

### Analysis of lncRNA sequencing data

**Figure 1:**
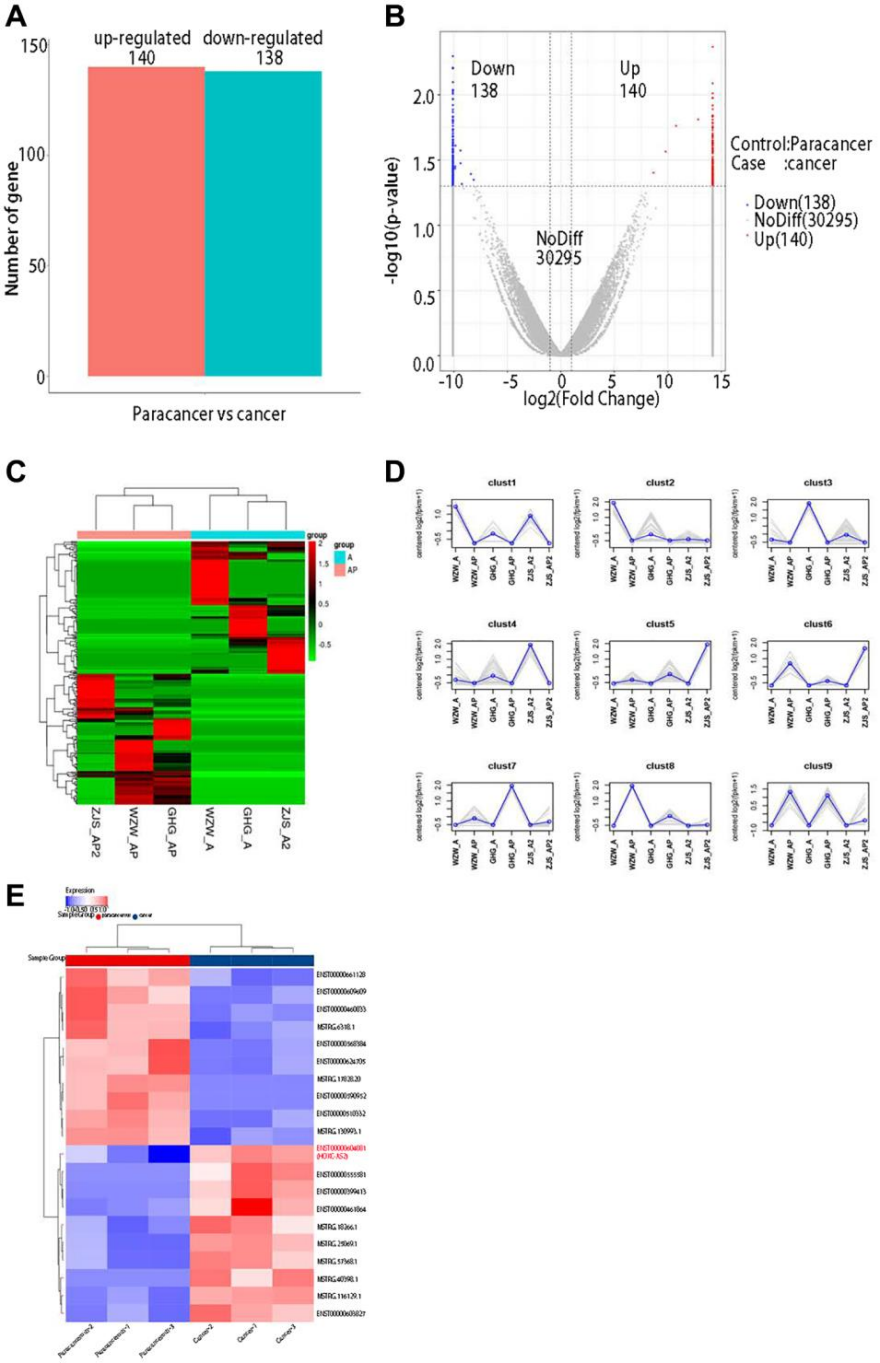
**lncRNA sequencing revealed abnormally high expression of HOXC-AS2 in hypopharyngeal cancer tissues.** (**A**) Statistical results of differentially expressed lncRNAs between different groups. Upregulated genes: lncRNAs upregulated in cases compared with controls; Downregulated genes: lncRNAs downregulated in cases compared with controls; Total DEGs: all differentially expressed lncRNAs in cases compared to controls. (**B**) Volcano plot of differentially expressed lncRNAs determined by sequencing in this study: The abscissa shows the log2-fold fold change values, and the ordinate shows the −log10 (*p*) values. The two vertical dashed lines in the figure indicate the differential expression thresholds (2-fold). The dotted line indicates the *P*-value threshold of 0.05. The red dots indicate upregulated lncRNAs in this group, the blue dots indicate downregulated lncRNAs in this group, and the gray dots indicate nonsignificant differentially expressed lncRNAs. (**C**) Clustering of differentially expressed lncRNAs: lncRNAs are shown in rows, each sample is shown in a column, red indicates high expression of a lncRNA, and green indicates low expression of a lncRNA. (**D**) Trend analysis after clustering in this study: the gray line shows the expression pattern of genes in each cluster, and the blue line shows the average expression level of all genes in the cluster in the sample. (**E**) Differential expression of lncRNAs was visualized on a heatmap after bioinformatics analysis of lncRNA sequencing data.

lncRNA sequencing was performed on 3 pairs of untreated primary hypopharyngeal carcinoma lesion tissues and adjacent normal tissues. First, DESeq was used to analyze the differential expression of lncRNAs after sequencing data were obtained. The screening criteria for differentially expressed genes were as follows: log2 (fold change) > 1 and *P* < 0.05. The results showed that 140 lncRNAs were significantly upregulated and 138 lncRNAs were significantly downregulated in cancer tissues compared with adjacent normal (*P* < 0.05, Figure 1A). In addition, the R software package ggplot2 was used to draw a volcano plot of the differentially expressed lncRNAs (Figure 1B). Next, we used the P-heatmap package in R software to conduct bidirectional clustering analysis on the union of differentially expressed genes and samples in all comparison groups. Clustering was performed according to the expression levels of the same lncRNA in different samples and the expression patterns of different lncRNAs in the same sample, and the Euclidean distance was calculated. Hierarchical clustering was performed with the maximum distance method (complete linkage) (Figure 1C). According to the results of the hierarchical clustering analysis described in the previous section, the differentially expressed genes were assigned to clusters according to their expression patterns (genes in the same cluster had similar expression trends) (Figure 1D). Considering the results of all these previous analyses, we finally generated a heatmap of the differentially expressed lncRNAs in R software and found that lncRNA HOXC-AS2 was the most highly expressed lncRNA in the sequencing data; thus, it was selected as the main research object of this study (Figure 1E). The top 10 upregulated lncRNAs were shown in Table 1. In summary, lncRNA sequencing was used to identify differentially expressed lncRNAs in three pairs of hypopharyngeal cancer tissues, and the results showed that HOXC-AS2 had the highest fold change; thus, we speculated that it might play an important role in the occurrence, development and metastasis of hypopharyngeal cancer.

**Table 1:** The top 10 upregulated lncRNAs.

**Gene Symbol**	**log_2_ fold change**	***P*-value**
HOXC-AS2	14.19	0.01
ENST00000555581	12.83	0.02
ENST00000399413	10.77	0.02
NCK1-AS1	9.80	0.03
MSTRG.18266.1	3.32	0.35
MSTRG.25069.1	3.10	0.38
MSTRG.57368.1	2.21	0.45
MSTRG.40398.1	2.02	0.47
MSTRG.116129.1	1.57	0.49
LINC00978	1.54	0.49

The genes in red are long non-coding RNA genes that have not been named.

### lncRNA HOXC-AS2 was upregulated in human hypopharyngeal carcinoma tissues and localized mainly in the cytoplasm

**Figure 2:**
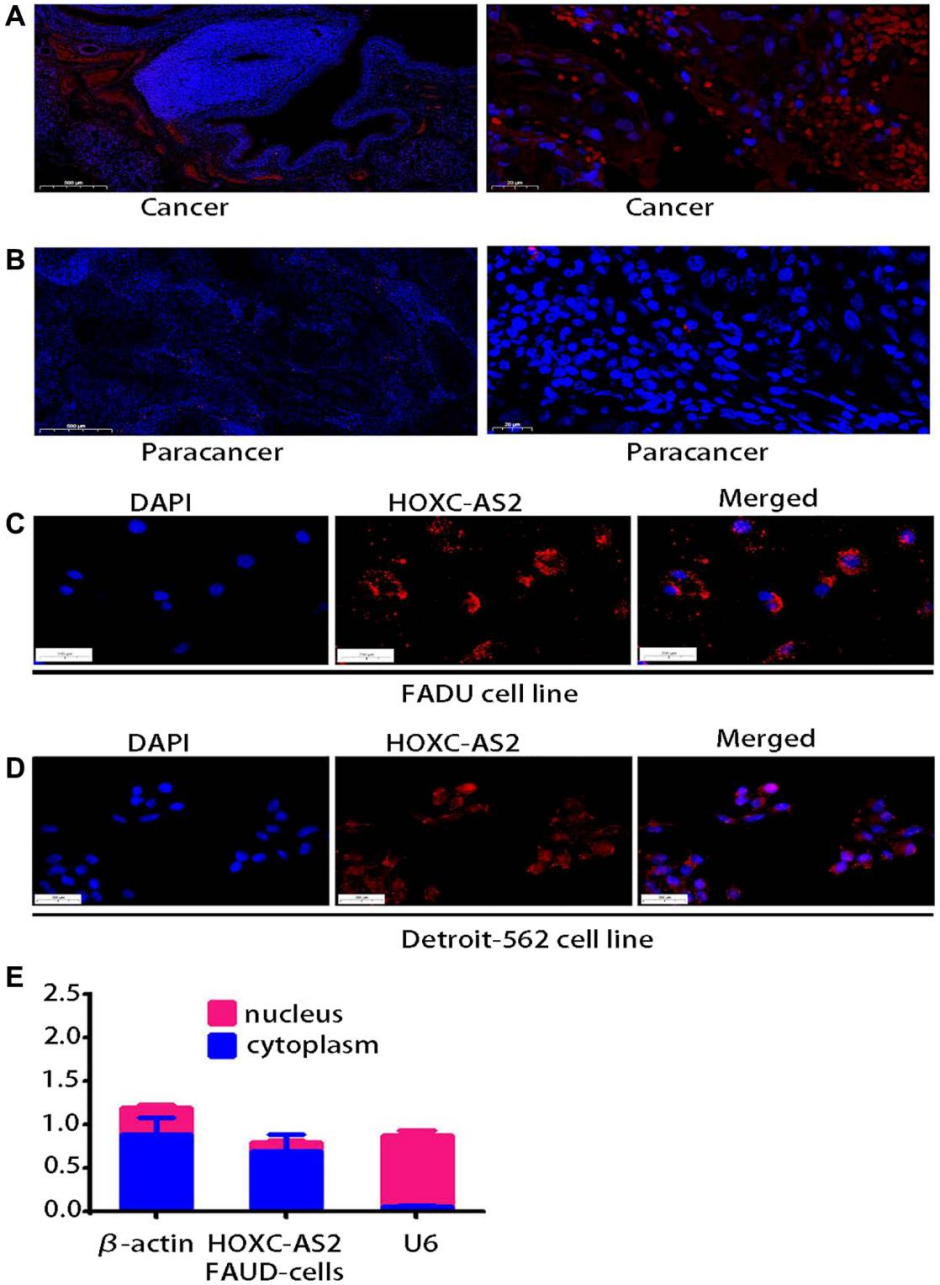
**lncRNA HOXC-AS2 was upregulated in human hypopharyngeal carcinoma tissues and localized mainly in the cytoplasm.** (**A**) Representative images of HOXC-AS2 expression in hypopharyngeal carcinoma tissues, as verified by FISH; the scale bars represent 500 μm and 20 μm, respectively. (**B**) Representative image of HOXC-AS2 expression in normal hypopharyngeal carcinoma-adjacent tissues, as verified by FISH; the scale bars represent 500 μm and 20 μm, respectively. (**C**) The intracellular localization of HOXC-AS2 was determined by RNA FISH in the FADU cell line. (**D**) The intracellular localization of HOXC-AS2 was determined by RNA FISH in the Detroit-562 cell line. (**E**) The nuclear and cytoplasmic distribution of HOXC-AS2 in the FADU cell line was evaluated by qRT-PCR.

The lncRNA sequencing results indicated that HOXC-AS2 was highly expressed in human hypopharyngeal cancer tissues. Therefore, we further verified the expression of HOXC-AS2 in 16 pairs of clinical samples by FISH. The expression of HOXC-AS2 in hypopharyngeal cancer tissues was significantly increased compared with that in normal tissues (Figure 2A, 2B). HOXC-AS2 was significantly more highly expressed in cancer tissues (9/10) compared with normal adjacent tissues in ten matched clinical specimens of hypopharyngeal cancer, and HOXC-AS2 expression was closely related to the lymph node metastasis status, TNM stage, and tumor size of hypopharyngeal cancer. However, due to the small number of cases, statistical analysis could not be performed. The function of a lncRNA is closely related to its subcellular localization. Thus, we performed FISH (Figure 2C, 2D) and subcellular fractionation assays (Figure 2E) in the FADU cell line to determine the localization of HOXC-AS2. In summary, the results of these experiments indicated that HOXC-AS2 is highly expressed in hypopharyngeal carcinoma and is localized mainly in the cytoplasm.

### HOXC-AS2 enhanced the viability, proliferation, invasion, and migration of hypopharyngeal carcinoma cells

**Figure 3:**
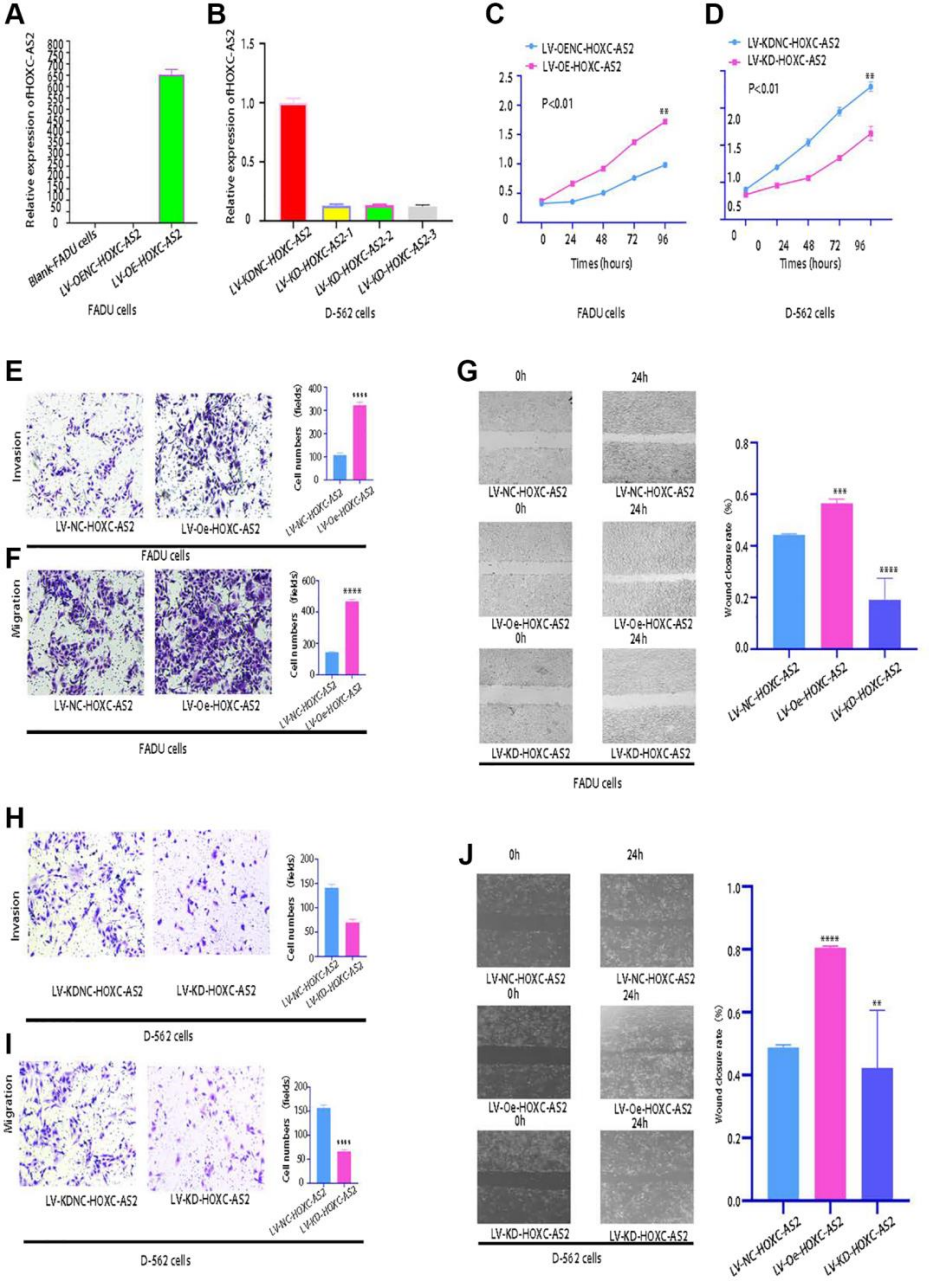
**HOXC-AS2 enhanced the viability, invasion, proliferation, and migration of hypopharyngeal cancer cells *in vitro* (magnification: 200×).** The data are shown as the means ± SEMs (*n* = 3); ^∗^*p* < 0.05, ^∗∗^*p* < 0.01, ^∗∗∗^*p* < 0.001, and ^∗∗∗∗^*p* < 0.0001. (**A**) The overexpression efficiency of LV-Oe-HOXC-AS2 and LV-OeNC-HOXC-AS2 in FADU cells was evaluated by qRT-PCR. (**B**) The knockdown efficiency of LV-KD-HOXC-AS2 and LV-KDNC-HOXC-AS2 in Detroit-562 cells was evaluated by qRT-PCR. (**C**) The viability of stable LV-Oe-HOXC-AS2-FADU and LV-OeNC-HOXC-AS2-FADU cell lines was evaluated by a CCK-8 assay. (**D**) The viability of LV-SH-HOXC-AS2-Detroit-562 and LV-SHNC-HOXC-AS2-Detroit-562 cells was evaluated by a CCK-8 assay. (**E**) The invasion ability of LV-Oe-HOXC-AS2-FADU and LV-OeNC-HOXC-AS2-FADU stable cells was evaluated by a Transwell assay. (**F**) The migration ability of LV-Oe-HOXC-AS2-FADU and LV-OeNC-HOXC-AS2-FADU stable cells was evaluated by a Transwell assay. (**G**) The migration ability of LV-Oe-HOXC-AS2-FADU and LV-OeNC-HOXC-AS2-FADU stable cells was evaluated by a wound healing assay. (**H**) The invasion ability of LV-SH-HOXC-AS2-Detroit-562 and LV-SHNC-HOXC-AS2-Detroit-562 stable cells was evaluated by a Transwell assay. (**I**) The migration ability of LV-KD-HOXC-AS2-Detroit-562 and LV-KDNC-HOXC-AS2-Detroit-562 stable cells was evaluated by a Transwell assay. (**J**) The migration ability of LV-SH-HOXC-AS2-Detroit-562 and LV-SHNC-HOXC-AS2-Detroit-562 stable cells was evaluated by a wound healing assay.

Analysis of the lncRNA sequencing data indicated that HOXC-AS2 had the highest expression level in hypopharyngeal cancer tissues, and further analysis of specimens from clinical cases also confirmed that HOXC-AS2 was highly expressed in hypopharyngeal cancer tissues. Therefore, we concluded that HOXC-AS2 plays a very important role in the progression of hypopharyngeal cancer. Gain- and loss-of-function experiments were then carried out to explore the biological functions of HOXC-AS2. Currently, there are only two hypopharyngeal cancer cell lines, namely, FADU and Detroit-562, and subsequent related experiments were performed in these two cell lines. A cell line with stable HOXC-AS2 overexpression was constructed in FADU cells and named LV-Oe-HOXC-AS2-FADU, and a cell line with stable HOXC-AS2 knockdown was constructed in Detroit-562 cells and named LV-SH-HOXC-AS2-Detroit-562. The HOXC-AS2 overexpression and knockdown efficiencies were verified through fluorescence microscopy and qRT-PCR. Lentiviral transduction was considered successful when more than 85% of cells exhibited green fluorescence 85% (Supplementary Figure 1A–1D). The qRT-PCR results showed that the HOXC-AS2 overexpression and knockdown efficiencies met the requirements for our project design (Figure 3A, 3B).

The viability, proliferation, migration and invasion of hypopharyngeal cancer cells were evaluated through CCK-8, Transwell, and wound healing experiments after HOXC-AS2 overexpression. Compared with LV-OeNC-HOXC-AS2 cells, hypopharyngeal cancer cells overexpressing HOXC-AS2 showed significant increases in viability (Figure 3C), invasion (Figure 3E) and migration (Figure 3F, 3G). However, compared with LV-SHNC-HOXC-AS2 cells, hypopharyngeal cancer cells with HOXC-AS2 knockdown showed significant decreases in viability (Figure 3D), invasion (Figure 3H), and migration (Figure 3I, 3J). The above data indicated that overexpression of HOXC-AS2 could significantly enhance the viability, proliferation, migration and invasion of hypopharyngeal cancer cells.

### HOXC-AS2 can bind to the SQSTM1 (P62) protein

**Figure 4:**
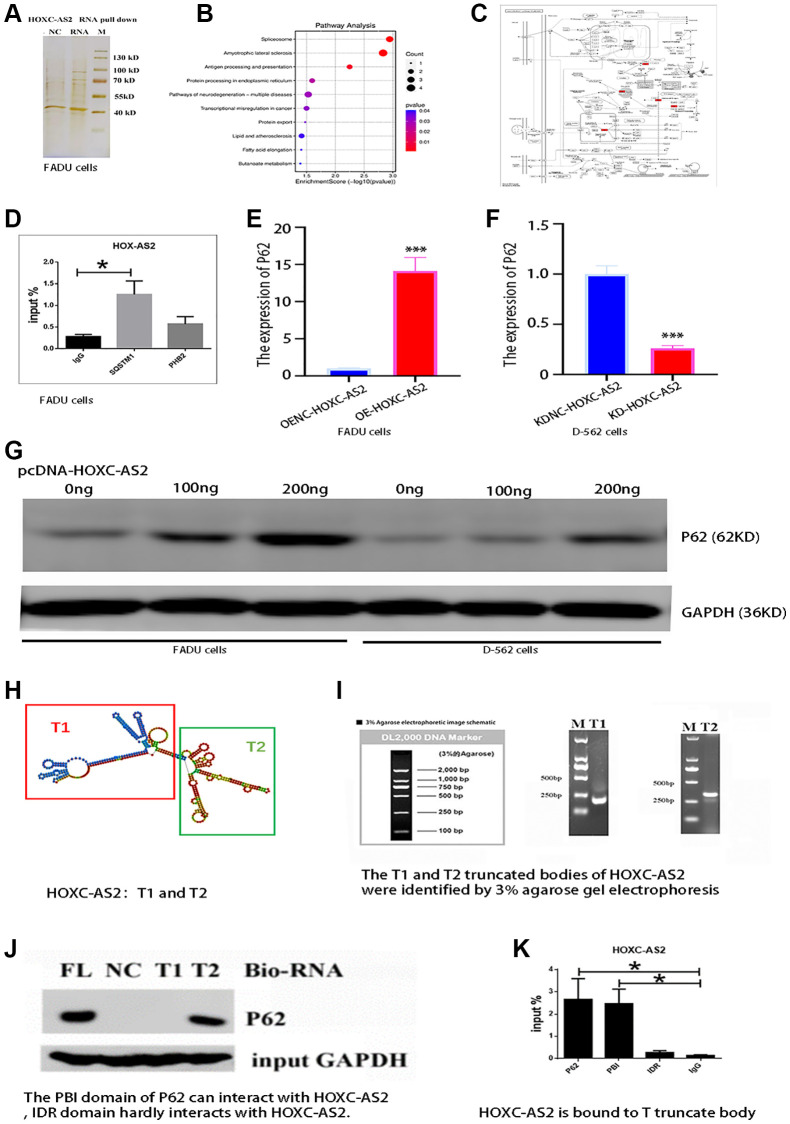
**HOXC-AS2 can bind to the SQSTM1 (P62) protein.** (**A**) Analysis of the RNA pulldown results by silver staining after HOXC-AS2 overexpression. (**B**) Bubble plots showing the results of the pathway analysis. (**C**) GO enrichment analysis of proteins identified in the HOXC-AS2 RNA pulldown assay. The P62 protein was found to be closely related to autophagy. (**D**) RIP assay of HOXC-AS2 with SQSTM1 and PHB2 proteins. (**E**) The mRNA expression level of P62 was confirmed through qRT-PCR analysis after HOXC-AS2 overexpression. (**F**) The mRNA expression level of P62 was confirmed through qRT-PCR analysis after HOXC-AS2 knockdown. (**G**) HOXC-AS2 overexpression plasmids were transfected into FADU and D-562 cells (0 ng, 100 ng and 200 ng), and changes in P62 protein expression were detected by western blot analysis. (**H**) Diagram of HOXC-AS2 gene body truncations: T1 and T2. (**I**) The T1 and T2 fragments of HOXC-AS2 were identified by 3% agarose gel electrophoresis. (**J**) The binding of HOXC-AS2 to the P62 protein was confirmed by a gene body truncation experiment. (**K**) The specific binding domain of HOXC-AS2 and P62 was confirmed by domain binding experiments.

The previous findings indicated that HOXC-AS2 plays a vital role in the formation and progression of hypopharyngeal cancer, but the related mechanism remains unclear and needs further exploration. Current studies have shown that lncRNAs can bind to DNA, RNA and proteins and regulate the expression of multiple genes at various levels, including the epigenetic, transcriptional and posttranscriptional levels, thus regulating the progression of diseases [20]. To further explore the mechanism of HOXC-AS2, we carried out RNA pulldown experiments and analyzed the precipitated proteins by mass spectrometry to explore the potential HOXC-AS2-binding proteins (Figure 4A). By MS analysis, 325 different proteins were found to binding potential with HOXC-AS2, and the top 20 proteins (DDX17, RBMX, ANXA2, TMPO, SQSTM1, FBRL, HNRNPL, NUP93, LMNB2, ADAR1, TAF15, HADHA, HSPA1A, DDX54, FUS, WDR46, CTNNA1, NOL6, HSPA5, DDX18) were manually selected (Supplementary Table 3). Then, through GO and KEGG pathway enrichment analyses, we found that HOXC-AS2 is involved mainly in the autophagy process in cells and regulates cell death. The results of protein profiling indicated that the PHB2 and SQSTM1 genes were related to mitochondrial autophagy; thus, these genes were selected as candidate targets for further research (Figure 4B, 4C). To confirm the evidence indicating that HOXC-AS2 binds to PHB2 and P62, we conducted a RIP assay. The RIP assay results showed that HOXC-AS2 and P62 could bind to each other, and the difference in binding was statistically significant compared with that in the IgG group (Figure 4D). Furthermore, the change in the mRNA expression level of SQSTM1 was evaluated in stable LV-Oe-HOXC-AS2-FADU and LV-OeNC-HOXC-AS2-FADU cells, and the mRNA expression level of SQSTM1 was found to be increased when HOXC-AS2 was overexpressed (Figure 4E). However, the mRNA expression of SQSTM1 was significantly downregulated when HOXC-AS2 was knocked down (Figure 4F). Moreover, we transfected the HOXC-AS2 overexpression plasmid into FADU and D-562 cells (0 ng, 100 ng and 200 ng) and then evaluated the changes in P62 protein expression. The protein expression level of P62 was significantly increased after HOXC-AS2 overexpression (Figure 4G). Furthermore, based on the prediction of RNAfold (RNAfold web server (Univia.ac.at)) software, we constructed gene body truncations of HOXC-AS2, dividing HOXC-AS2 into the T1 and T2 segments (Figure 4H). Then, the constructed HOXC-AS2 plasmid was used as a template for PCR amplification. The primer sequences targeting the T1 and T2 gene body segments are detailed in Supplementary Table 2. The PCR products were analyzed by 3% agarose gel electrophoresis, and the target fragments were identified. The T1 and T2 target fragments contained 237 bp and 285 bp, respectively. The agarose gel images used for identification of the segments are shown in Figure 4I. Then, the product was recovered from the agarose gel and purified on a column. The RNA pulldown assay was then carried out according to the instructions of the RNA pulldown kit. The measured concentrations of the obtained protein products were as follows: HOXC-AS2 FL, 0.2 μg/μl; HOXC-AS2 T1, 0.25 μg/μl; HOXC-AS2 T1, 0.27 μg/μl; control, 0.19 μg/μl. The protein products were verified by western blot analysis, and the results suggested that the T2 segment of HOXC-AS2 could interact with SQSTM1 (Figure 4J). Next, we continued to explore the interaction domains of HOXC-AS2 and SQSTM1 by RIP experiments. We constructed the pcDNA3.1-SQSTM1 plasmid, pcDNA3.1-PBI plasmid and pcDNA3.1-IDR plasmid based on the domains of SQSTM1 and transfected them into FADU cells. A RIP assay was performed on cells collected 24 h after transfection. SQSTM1 and its PBI domain could interact with HOXC-AS2, while the IDR domain hardly interacted with HOXC-AS2 (Figure 4K). Through the above experiments and data, we confirmed the specific binding site of HOXC-AS2 and SQSTM1 for the first time and explored the mechanism of action of HOXC-AS2 in depth. In conclusion, these results indicate that HOXC-AS2 can bind to the SQSTM1 protein.

### HOXC-AS2 activated the NF-kB signaling pathway by binding to the SQSTM 1 protein

**Figure 5:**
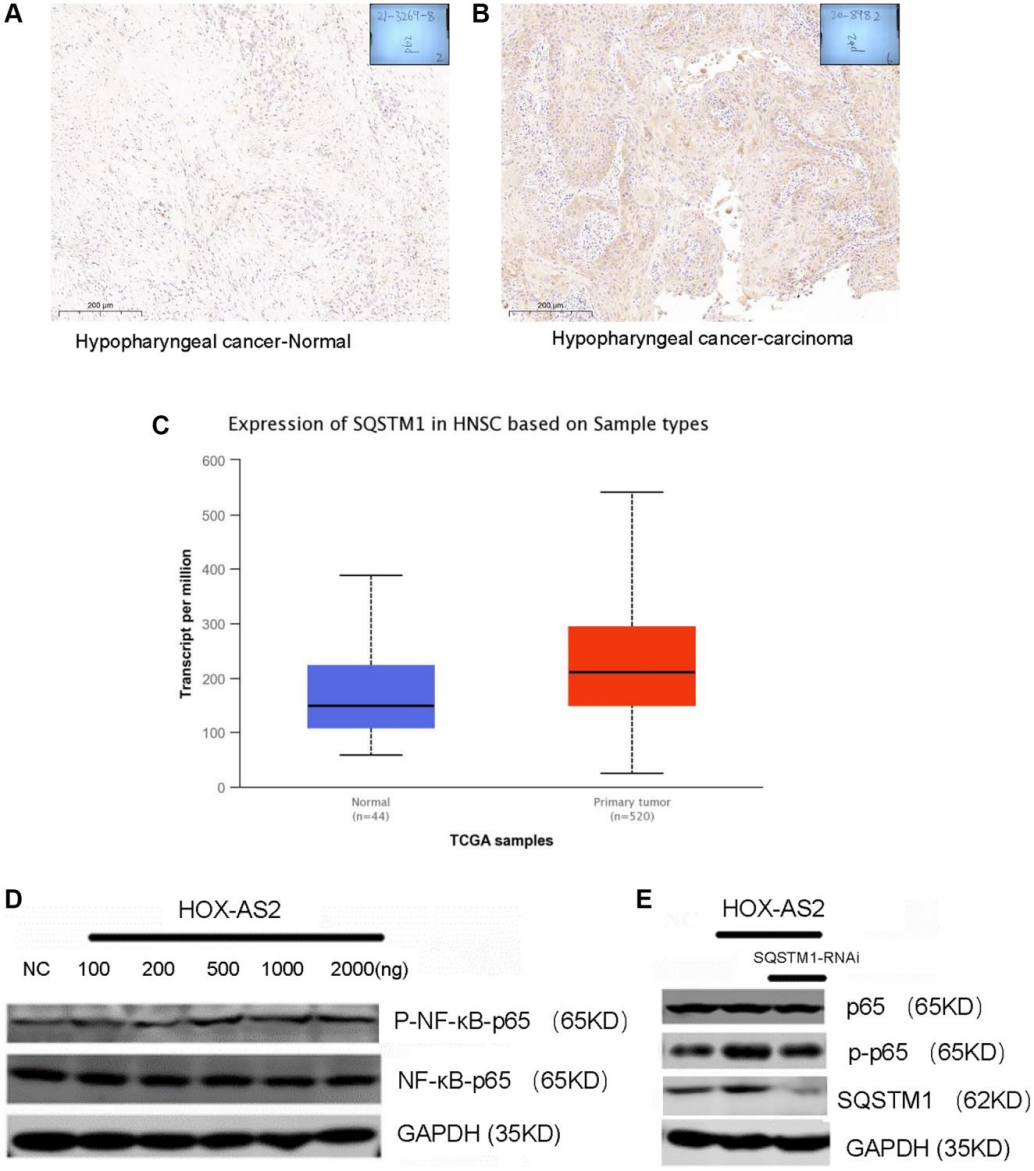
**HOXC-AS2 activated the NF-kB signaling pathway by binding to the SQSTM 1 protein.** (**A**) Representative image of P62 protein expression in normal hypopharyngeal carcinoma-adjacent tissue, as evaluated by IHC staining. (**B**) Representative image of P62 protein expression in hypopharyngeal carcinoma tissue, as evaluated by IHC staining. (**C**) The expression level of P62 in head and neck squamous cell carcinoma was confirmed by TCGA database analysis. (**D**) Activation of the NF-KB signaling pathway after HOXC-AS2 overexpression was detected by western blot analysis. (**E**) The protein expression level of NF-KB was measured by western blot analysis after P62 was knocked down in HOXC-AS2-overexpressing cells.

SQSTM1 is an important selective autophagy adaptor protein that contains 6 functional domains, including a ubiquitin-related domain; a Kelch-like ECH-associated protein domain; a microtubule-related protein 1A/1B light chain 3 domain; a tumor necrosis factor receptor-related factor 6, Phox and Bem1p domain; and a zinc finger region. SQSTM1 plays an important role in tumor formation by regulating signaling pathways such as the nuclear transcription-related factor 2-antioxidant response, NF-κB and caspase 8-mediated apoptosis pathways [21–23]. Based on the important biological function of SQSTM1, we first explored the expression of SQSTM1 in hypopharyngeal carcinoma tissues through immunohistochemical (IHC) analysis. SQSTM1 was highly expressed in hypopharyngeal carcinoma tissues compared with normal tissues (Figure 5A, 5B). Then, we further verified the expression level of SQSTM1 in head and neck malignancies in the TCGA database. Compared with that in normal tissues, the expression level of SQSTM1 in HNSC tissues was significantly increased (*P* < 0.01, Figure 5C). The above data indicate that SQSTM1 is highly expressed in hypopharyngeal cancer and is involved in its formation and progression. Next, we studied whether HOXC-AS2 binding to the SQSTM1 protein can activate the downstream NF-kB signaling pathway to exert its biological effects. To this end, we transfected the HOXC-AS2 overexpression plasmid into FADU cells and collected the cells to measure the expression of the NF-KB protein by western blot analysis. The results demonstrated that overexpression of HOXC-AS2 can activate the NF-κB signaling pathway (Figure 5D). Thus, we next sought to prove that SQSTM1 plays a key role in the activation of the NF-KB signaling pathway. We transfected the HOXC-AS2 overexpression plasmid and SQSTM1 knockdown plasmid into FADU cells, collected the cells and performed western blot analysis. The results showed that SQSTM1 knockdown significantly affected the activation of the NF-κB signaling pathway by HOXC-AS2 (Figure 5E). In summary, we found that HOXC-AS2 can activate the downstream NF-KB signaling pathway after binding to the SQSTM1 protein, thus regulating autophagy.

### HOXC-AS2 affected the expression of HMOX1 by regulating the NF-KB signaling pathway and ultimately regulated autophagy in hypopharyngeal cancer cells

**Figure 6:**
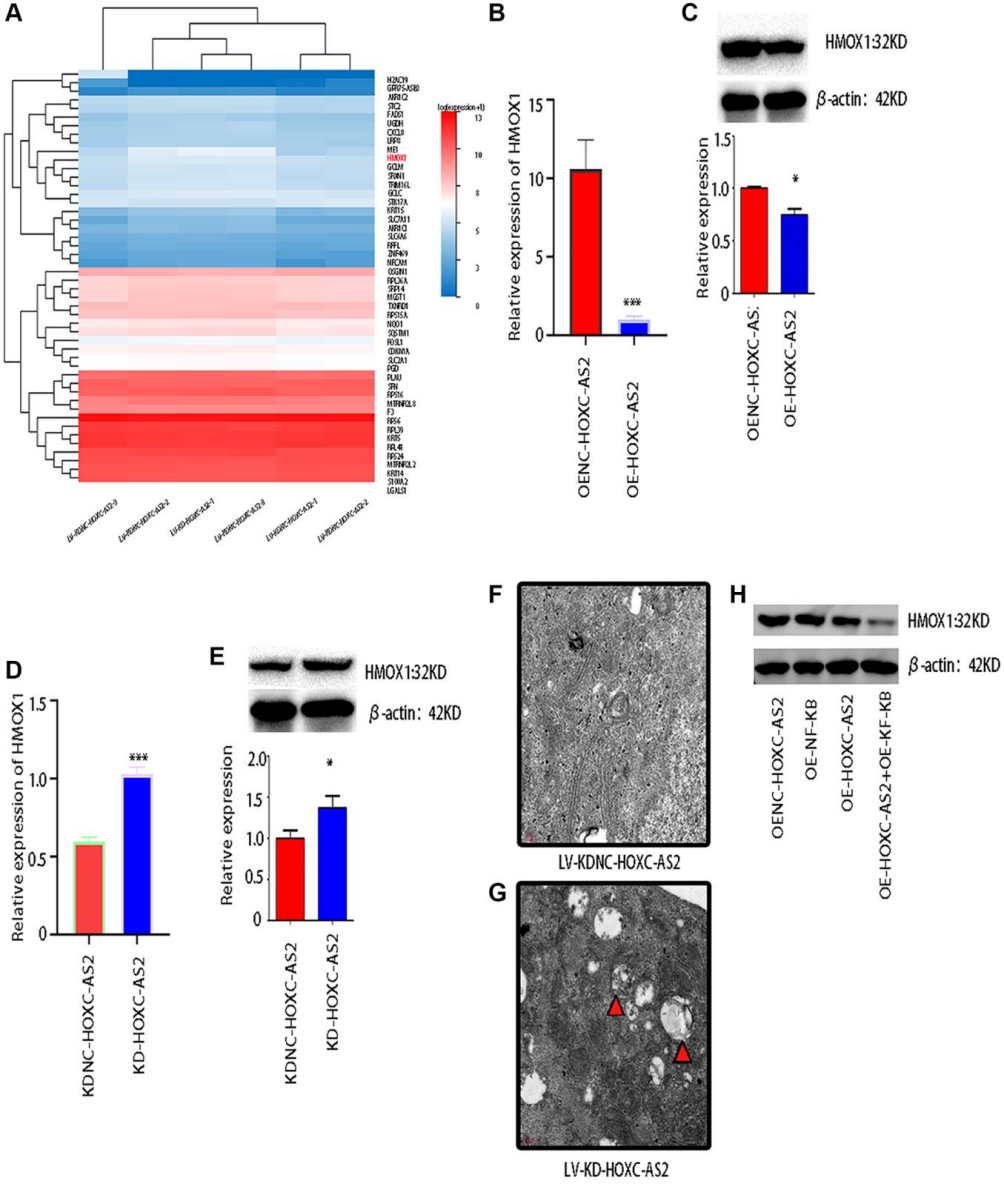
**HOXC-AS2 affected the expression of HMOX1 by regulating the NF-KB signaling pathway and ultimately regulated autophagy in hypopharyngeal cancer cells.** (**A**) Transcriptome sequencing was performed after HOXC-AS2 knockdown, and the heatmap shows the differentially expressed genes identified by sequencing. NC-transfected cells were used as controls. (**B**) The mRNA expression level of HMOX1 was confirmed through qRT-PCR analysis after HOXC-AS2 overexpression. (**C**) The protein expression level of HMOX1 was confirmed through western blot analysis after HOXC-AS2 overexpression. (**D**) The mRNA expression level of HMOX1 was confirmed through qRT-PCR analysis after HOXC-AS2 knockdown. (**E**) The protein expression level of HMOX1 was confirmed through western blot analysis after HOXC-AS2 knockdown. (**F**, **G**) Autophagosome formation in hypopharyngeal cancer cells after HOXC-AS2 knockdown was observed by transmission electron microscopy, and NC-transfected cells were used as controls. (**H**) The protein expression level of HMOX1 was affected by HOXC-AS2 and NF-KB.

To explore the common downstream target genes of HOXC-AS2 and NF-KB involved in regulating autophagy, we knocked down HOXC-AS2 in Detroit-562 cells, and NC-transfected cells were used as the controls. Then, transcriptome sequencing was performed on the two groups of samples. Next, we performed bioinformatics analysis on the obtained data to screen for differentially expressed genes. HMOX1, AKR1C2 and other genes were statistically significantly differentially expressed; among all the identified differentially expressed genes, HMOX1 was the most statistically significant (Figure 6A). Next, we verified the expression of HMOX1 after HOXC-AS2 overexpression by qRT-PCR and western blot analysis and found that the mRNA and protein levels of HMOX1 were decreased when HOXC-AS2 was overexpressed (*P* < 0.05, Figure 6B, 6C). In contrast, when HOXC-AS2 was knocked down, the mRNA and protein levels of HMOX1 were significantly increased (*P* < 0.05, Figure 6D, 6E). To further confirm whether HOXC-AS2 regulates the progression of hypopharyngeal cancer by regulating autophagy, we knocked down HOXC-AS2 in Detroit-562 cells and observed the formation of autophagosomes by transmission electron microscopy. NC-transfected cells were used as controls. After HOXC-AS2 knockdown, the number of autophagosomes formed was significantly greater than that in the control group (Figure 6F, 6G). In summary, we confirmed that HOXC-AS2 can affect the expression of HMOX1 by regulating the NF-KB signaling pathway, thus regulating autophagy and the progression of hypopharyngeal cancer (Figure 6H).

### Animal experiments

**Figure 7:**
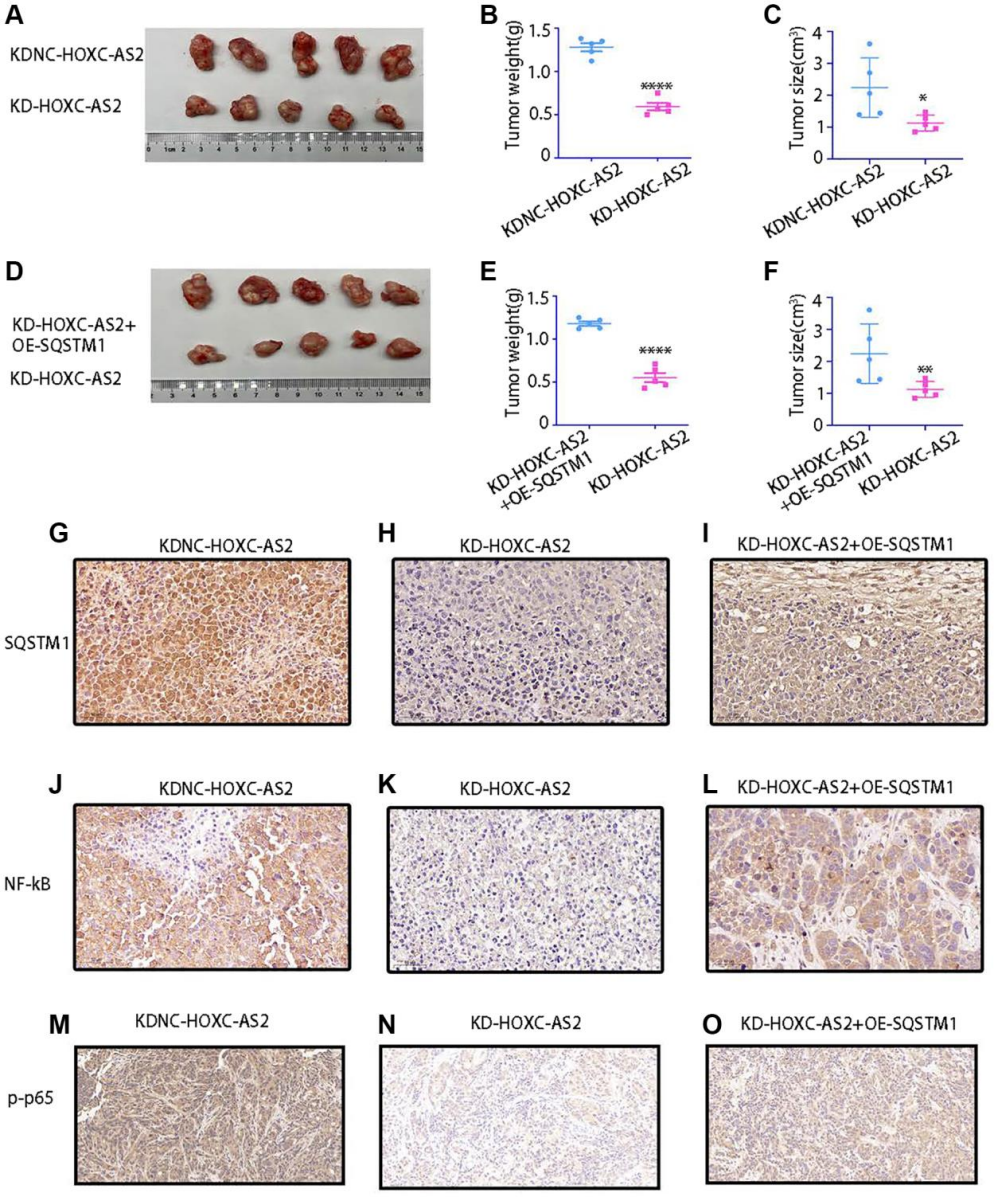
**The results of animal experiments confirmed that HOXC-AS2 can promote hypopharyngeal cancer progression.** (**A**–**C**) Morphological characteristics of subcutaneous xenografts (**A**), tumor weight (**B**), and tumor size (**C**) on day 35 in mice injected with LV-KDNC-HOXC-AS2-D-562 cells and LV-KD-HOXC-AS2-D-562 cells (five mice per group). (**D**–**F**) Morphological characteristics of subcutaneous xenografts (**D**), tumor weight (**E**), and tumor size (**F**) on day 35 in mice injected with LV-KD-HOXC-AS2 +OE-P62-D-562 cells and LV-KD-HOXC-AS2-D-562 cells (five mice per group). (**G**–**I**) IHC analysis of P62 protein expression in tumors from the LV-KDNC-HOXC-AS2, LV-KD-HOXC-AS, and LV-KD-HOXC-AS2 +OE-P62 groups. (**J**–**L**) IHC analysis of NF-kB protein expression in tumors from the LV-KDNC-HOXC-AS2, LV-KD-HOXC-AS, and LV-KD-HOXC-AS2 +OE-P62 groups. (**M**–**O**) IHC analysis of p-p65 protein expression in tumors from the LV-KDNC-HOXC-AS2, LV-KD-HOXC-AS, and LV-KD-HOXC-AS2 +OE-P62 groups.

Based on the results of the previous experiments, we further explored the role of HOXC-AS2 in hypopharyngeal carcinoma through animal experiments. To this end, we established a subcutaneous tumorigenesis model in nude mice. Four- to six-week-old mice were divided into four groups: the LV-KDNC-HOXC-AS2 and LV-KD-HOXC-AS2 groups and the LV-KD-HOXC-AS2+OE-SQSTM1 and KD-HOXC-AS2 groups (*n* = 5 mice per group). Then, Detroit-562 cells were cultured according to the experimental group design, and these cells (6 × 10^5^) were resuspended in 100 μl of DMEM and injected into the flanks of mice. Two weeks later, tumor formation was observed and recorded, and the tumor volume was calculated using the following equation: volume = 1/2 × (width2 × length). After five weeks, the mice were euthanized, and subcutaneous tumors were harvested. Compared with those in the control group, the volume and weight of tumors were significantly reduced in mice injected with cells with HOXC-AS2 knockdown (Figure 7A–7C). However, compared with that in the HOXC-AS2 knockdown group, the tumorigenic ability of HOXC-AS2 was restored when SQSTM1 was overexpressed in the cells with stable HOXC-AS2 knockdown (Figure 7D–7F). In addition, subcutaneous tumors were harvested from mice in the KDNC-HOXC-AS2, KD-HOXC-AS2, and KD-HOXC-AS2+OE-HOXC-AS2 groups, and the protein expression of SQSTM1 and NF-KB was evaluated by IHC staining. SQSTM1, NF-KB and p-p65 protein expression was decreased in the HOXC-AS2 knockdown group (Figure 7G, 7H, 7J, 7K, 7M, 7N). However, upon SQSTM1 overexpression, the protein expression of SQSTM1, NF-KB and p-p65 in the HOXC-AS2 knockdown group were partially restored (Figure 7I, 7L and 7O). In conclusion, HOXC-AS can exert its biological effect and regulate the progression of hypopharyngeal cancer by binding to SQSTM1 to regulate NF-KB expression.

## DISCUSSION

HSCC is the most malignant type of head and neck cancer, with a 5-year survival rate of 30%–50% [24]. HSCC is characterized by a high incidence of lymph node metastasis, and the overall survival rate of patients who develop lymph node metastasis is decreased by at least 25% [25].

lncRNAs are transcripts with a length of more than 200 nucleotides and limited or no protein-coding ability [26]. lncRNAs exist in either the nucleus or cytoplasm, and the functions of lncRNAs in these locations are also quite different. Cytoplasmic lncRNAs, whose role is to mediate signal transduction pathways, translation programs, and posttranscriptional control of gene expression, compete for binding to miRNAs [27] and proteins [28] to regulate their activity and levels, influence posttranslational modifications [29], or mediate mRNA translation and stability [30]. Current studies have shown that lncRNAs play a key role in the occurrence, development and metastasis of hypopharyngeal cancer [11, 12, 31, 32]. For example, HOXA11-AS is upregulated in hypopharyngeal cancer and acts on MiR-155 through a ceRNA mechanism to promote the progression of hypopharyngeal cancer [12]. However, exploration of the mechanism of lncRNAs in hypopharyngeal cancer is still very limited. Our research group conducted lncRNA sequencing on 3 pairs of hypopharyngeal cancer patient tumor and adjacent normal tissues and found that 140 lncRNAs were upregulated and 138 lncRNAs were downregulated. Among the lncRNAs with upregulated expression, HOXC-AS2 had the highest fold change in expression. Therefore, HOXC-AS2 was selected as the research object in this study.

The host gene of HOXC-AS2 is located on chromosome 12q13.13. Studies have shown that HOXC-AS2 regulates the proliferation, apoptosis and migration of NSCLC cells by binding to the HOXC13 gene. In this paper, the authors confirmed that HOXC-AS2 can regulate apoptosis and EMT in NSCLC cells, thus promoting the formation, progression and metastasis of NSCLC [14]. HOXC-AS2 also regulates endometrial cancer progression, a finding that demonstrates that HOXC-AS2 can play a cancer-promoting role through a ceRNA mechanism. Specifically, the HOXC-AS2/miR-876-5p/HKDC1 axis can regulate pyroptosis in endometrial cancer cells, especially in patients with diabetes [13], and EMT in glioma cells [33]. Based on the current literature, we determined that HOXC-AS2 is an oncogene that can regulate apoptosis, EMT and pyroptosis in tumor cells. However, other aspects of its function need further exploration. To date, the role of HOXC-AS2 in hypopharyngeal carcinoma has not been explored. Therefore, here, a lncRNA (HOXC-AS2) closely related to hypopharyngeal cancer was accurately identified through sequencing of clinical case samples and bioinformatics analysis. Then, through verification assays in cell lines and by FISH, we found that in addition to its known function, HOXC-AS2 is upregulated in hypopharyngeal cancer and primarily localized in the cytoplasm of hypopharyngeal cancer cells. Thus, we speculated that HOXC-AS2 also plays an important role in the progression of hypopharyngeal cancer. The best way to study the function of a gene is to overexpress it or knock it down and determine how these modulations change the biological functions of cells. After overexpression and knockdown of HOXC-AS2, we found that overexpression of HOXC-AS2 enhanced the viability, proliferation and migration of hypopharyngeal cancer cells and that these biological properties of hypopharyngeal cancer cells were correspondingly inhibited when HOXC-AS2 was knocked down. Since lncRNAs have no intrinsic protein-coding ability, they need to bind to other RNA-binding proteins to perform their functions. Thus, we conducted RNA pulldown experiments, and the protein products were identified by MS analysis. We identified 325 proteins that interact with HOXC-AS2. Then, we found that HOXC-AS is closely related to autophagy through GO enrichment analysis. Then, two genes, PHB2 and P62, were identified through experimental verification and overlap analysis. Finally, it was determined through a RIP assay that HOXC-AS2 is most closely related to P62. Therefore, we first demonstrated that HOXC-AS2 can bind to the P62 protein to regulate autophagy in hypopharyngeal cancer cells. We explored the binding domain of HOXC-AS2 to the P62 protein in depth and found that SQSTM1 and its PBI domain can interact with HOXC-AS2, while the IDR domain hardly interacted with HOXC-AS2. Furthermore, we found that HOXC-AS2 can activate the downstream NF-KB signaling pathway after binding to the SQSTM1 protein, thus regulating autophagy. To further explore the NF-KB-regulated autophagy genes, we performed transcriptome sequencing and bioinformatics analysis. Our results confirmed that HOXC-AS2 can affect the expression of HMOX1 by regulating the NF-KB signaling pathway, thus regulating autophagy and the progression of hypopharyngeal cancer. In this study, we found by transmission electron microscopy that autophagosome formation in hypopharyngeal cancer cells was significantly increased when HOXC-AS2 was knocked down, a finding that provided expanded evidence supporting the results of our subcellular localization experiment. Finally, we verified in animal experiments that HOXC-AS2 can indeed promote the formation and progression of hypopharyngeal cancer and that the P62 protein plays a decisive role in this process.

In conclusion, HOXC-AS2 can bind to the p62 protein to activate the NF-KB signaling pathway, thereby regulating the expression of HMOX1 and ultimately affecting autophagy in hypopharyngeal cancer cells to facilitate the formation and progression of hypopharyngeal cancer.

## Supplementary Materials

Supplementary Figure 1

Supplementary Table 1

Supplementary Table 2

Supplementary Table 3
