# Supplementary material for: lncRNA HOXC-AS2 promotes the progression of hypopharyngeal cancer by binding to the P62 protein mediating the autophagy process
Source: Aging (Albany NY). 2023 Nov 8;15(21):12476–96. doi: 10.18632/aging.205192 (PMC10683610; doi:10.18632/aging.205192)
Supplement: Supplementary Figure 1 [file aging-15-205192-s001.pdf]

SUPPLEMENTARY FIGURE

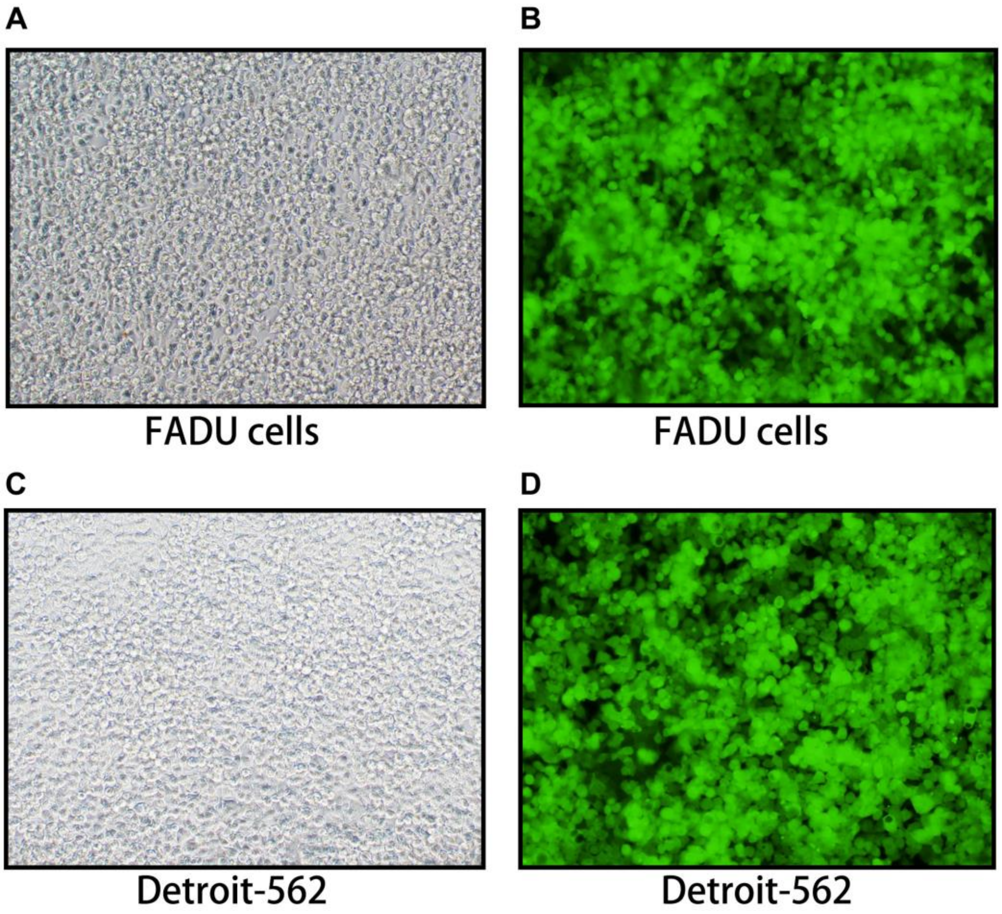

Supplementary Figure 1. The transfection efficiency of cells transfected with Lentivirus under fluorescence microscope. (**A**) Clear field of FADU cells not infected with HOXC-AS2 overexpressing lentivirus, magnification 200×. (**B**) Green fluorescence field after infection of FADU cells by lentivirus overexpressing HOXC-AS2. Lentivirus infection rate exceeds 85%, magnification 200×. (**C**) Clear field of Detroit-562 cells uninfected with HOXC-AS2 knockdown lentivirus, magnification 200×. (**D**) Green fluorescence field after infection of Detroit-562 cells with lentivirus knockdown HOXC-AS2, lentivirus infection rate was over 85%, magnification 200×.
