# Supplementary material for: lncRNA HOXC-AS2 promotes the progression of hypopharyngeal cancer by binding to the P62 protein mediating the autophagy process
Source: Aging (Albany NY). 2023 Nov 8;15(21):12476–96. doi: 10.18632/aging.205192 (PMC10683610; doi:10.18632/aging.205192)
Supplement: Supplementary Table 1 [file aging-15-205192-s002.pdf]

SUPPLEMENTARY TABLE

Supplementary Table 1. Clinicopathologic characteristics of 3 patients with lncRNA sequencing.

Number	Gender	Age (years)	T stage	LM	DM	Tumor size (cm)	Smoking history	Drinking history	Family history	Pathological pattern
1	Male	69	T4	N3	No	5.9 × 4.7 × 4.3	Yes	Yes	No	Poor differentiation squamous cell carcinoma, individual vascular invasion
2	Male	57	T3	N2	No	4.5 × 4.0 × 3.8	Yes	Yes	No	Well differentiated squamous cell carcinoma
3	Male	57	T3	N1	No	3.7 × 3.2 × 3.0	Yes	Yes	No	Moderately differentiated squamous cell carcinoma, Invasion of the surrounding muscle tissue
